# Breastfeeding Determinants in Bafia Health District (Cameroon): A Cross‐Sectional Study

**DOI:** 10.1155/ijpe/8888854

**Published:** 2026-07-30

**Authors:** Dominique Enyama, Cavin Epie Bekolo, Célestin Géraud Yamb Essola, Diomède Noukeu Njinkui, Marie Christine Atyam Ekoto, Sime Tchouamo Arielle Annick, Jérôme Ateudjieu

**Affiliations:** ^1^ Department of Pediatrics, Child and Adolescent Health, Faculty of Medicine and Pharmaceutical Sciences, University of Dschang, Dschang, Cameroon, univ-dschang.org; ^2^ Department of Pediatrics and Subspecialties, Douala Gyneco-Obstetric and Pediatric Hospital, Douala, Cameroon; ^3^ Department of Pediatrics, Dschang Regional Hospital Annex, Dschang, Cameroon; ^4^ Department of Pediatrics, Yaoundé Gyneco-Obstetric and Pediatric Hospital, Yaoundé, Cameroon

**Keywords:** associated factors, Cameroon, early initiation, exclusive breastfeeding

## Abstract

Exclusive breastfeeding (EBF) provides optimal nutrition, but only 48% of Cameroonian infants are breastfed early within 1 h after birth, and 40% are EBF until 6 months, below WHO′s 2025 target of 50%. We aimed to identify factors associated with early breastfeeding initiation and exclusivity in Bafia Health District, Cameroon. A cross‐sectional study at six health facilities examined 422 newborns (≤ 14 days) for early initiation and 544 infants (6–15 months) for EBF. Data from structured questionnaires on sociodemographic, obstetric, and breastfeeding factors were analyzed using multivariate binary logistic regression. The prevalence of early breastfeeding initiation was 73.7% (95% CI: 69.30%–77.67%), and EBF prevalence was 53.9% (95% CI: 49.66%–58.01%). Factors promoting early initiation included vaginal delivery (aOR = 5.64), maternal pregnancy awareness (aOR = 2.22), and singleton pregnancy (aOR = 2.31). Continued EBF was positively influenced by maternal pregnancy awareness (aOR = 2.67), lack of maternal employment (aOR = 7.23), maternal education (aOR = 2.04), and Muslim faith (aOR = 4.20). Although breastfeeding rates are higher than the national average, they still fall short of WHO recommendations. Enhancing maternal education and awareness initiatives during pregnancy could greatly boost early initiation and EBF duration, leading to improved infant health outcomes.

## 1. Introduction

Breastfeeding involves feeding a baby with either expressed or natural breast milk, and it benefits both mother and child by supporting growth, strengthening bonds, boosting immunity, and reducing expenses related to substitutes and healthcare [1]. Around 10.9 million children under five die each year worldwide, with 35% dying before their first birthday, mainly due to preventable malnutrition that could be avoided through exclusive breastfeeding (EBF) [2]. If practiced universally, breastfeeding could save 1.2 million lives annually [3]. Since 2001, the WHO has recommended early initiation of breastfeeding within 1 h of birth, EBF for the first 6 months, and continued breastfeeding with complementary foods up to 2 years or beyond [4]. A meta‐analysis from four countries shows that early breastfeeding reduces the risk of neonatal death by 33% [5]. Delaying initiation increases the risk of infections and respiratory issues in infants [6]. Despite these benefits and WHO guidelines, breastfeeding rates remain low, with only 20% of infants under 6 months being exclusively breastfed in West and Central Africa [7]. The 2018 Cameroon survey reports that 92% of children are breastfed, but only 48% start within an hour, and 40% are exclusively breastfed for 6 months. The average breastfeeding duration is 15.3 months [8].

Several factors have been shown to influence breastfeeding practices. In Canada, low maternal educational level, marital status, and mode of delivery were identified as determinants of EBF [9]. These findings underscore the importance of sociodemographic and obstetric factors in shaping breastfeeding behaviors. A 2018 study by Mukuku in the Democratic Republic of Congo demonstrated that first‐born birth order and delayed initiation of breastfeeding were significantly associated with early cessation of EBF, highlighting the importance of timely support in the immediate postpartum period [10]. A study by Agho et al. in Nigeria found that mothers employed outside the home were significantly less likely to maintain EBF, largely due to the constraints of early return to work, separation from the infant, and insufficient workplace provisions for breastfeeding [11].

Although previous studies have investigated EBF determinants in Cameroon, including in urban settings such as Yaoundé [12], no study to date has simultaneously examined early breastfeeding initiation and EBF exclusivity in a semirural district‐level context such as Bafia Health District. In Cameroon, despite governmental efforts and initiatives by health professionals, breastfeeding rates remain suboptimal: The 2018 national survey reports that only 40% of newborns are exclusively breastfed, and only 48% are breastfed within the first hour of birth [8]. Data on breastfeeding determinants at the district level remain scarce, justifying the present study. This study is therefore aimed at identifying the factors associated with the early initiation and continuation of EBF in the Bafia Health District, addressing an important geographical and methodological gap in the Cameroonian breastfeeding literature.

## 2. Methods

### 2.1. Study Design

We conducted a descriptive cross‐sectional study with analytical components examining sociodemographic factors, knowledge, and practices related to breastfeeding in the Bafia Health District.

### 2.2. Setting

The study was conducted over a period of 10 months, from October 1, 2022, to June 30, 2023, in six health facilities within Bafia Health District, Cameroon, approximately 120 km north of Yaoundé. Health facilities were selected using purposive nonprobabilistic sampling based on the following criteria: being a functional public or private health facility within the Bafia Health District, providing both maternity and vaccination services, and maintaining a monthly delivery volume sufficient for participant recruitment. The six selected facilities included two district hospitals, three health centers, and one confessional clinic, collectively serving the Bafia Health District catchment population. The study included mothers and newborns in the immediate postpartum period and up to 14 days later, as well as mother–infant pairs aged 6–15 months attending vaccination appointments.

### 2.3. Participants

We included 422 newborns for early initiation and 544 infants for EBF. Inclusion criteria consisted of mothers with healthy newborns and infants within specific age ranges attending selected health facilities. Exclusions were applied to mothers with incomplete medical records and those unable to give informed consent.

Sample size was calculated using the Cochran formula: *n* = *Z* ^2^ × *p*(1 − *p*)/*e* ^2^, where *Z* = 1.96 (95% confidence level), *p* = 0.40 (national EBF prevalence [9]), and *e* = 0.05 (margin of error), yielding a minimum of 369 participants. Adjusted for a 10% nonresponse rate, the target sample was 406 per outcome group. Participants were enrolled using consecutive nonprobabilistic sampling at each health facility during the study period. The achieved samples of 422 newborns and 544 infants exceeded the calculated minimums.

#### 2.3.1. Outcome Variables

Early initiation of breastfeeding was defined as the initiation of breastfeeding within 1 h of birth, as per WHO criteria [4]. This was assessed in newborns aged 0–14 days. EBF was defined as feeding the infant only breast milk (including expressed breast milk), with no other liquids or solids except for oral rehydration salts, drops, or syrups (vitamins, minerals, and medicines) [4]. This was assessed by 24‐h dietary recall in infants aged 6–15 months.

### 2.4. Data Collection Procedure

Data were collected through face‐to‐face structured interviews using a pretested questionnaire administered by trained research assistants. The questionnaire was pretested on 30 participants not included in the main study, and necessary adjustments were made prior to data collection. The questionnaire collected data on sociodemographic characteristics (such as maternal age, education, occupation, religion, and marital status), obstetric factors (including parity, delivery mode, pregnancy type, and antenatal care), infant features (birth weight, gestational age, and sex), and breastfeeding practices and maternal awareness.

### 2.5. Statistical Analysis

Data were entered into Excel spreadsheets and exported to SPSS v22 for analysis. Descriptive statistics presented variables as percentages with 95% confidence intervals and means ± standard deviation. Multivariate binary logistic regression identified factors associated with early initiation and EBF exclusivity. The following covariates were included in the multivariate models based on biological plausibility and statistical significance at univariate analysis (*p* < 0.20): maternal age, marital status, level of education, monthly income, religion, occupation, parity, mode of delivery, pregnancy type, antenatal care attendance, gestational age, birth weight, and maternal breastfeeding awareness. Statistical significance was set at *p* < 0.05.

### 2.6. Ethics

The study received approval from the Centre Regional Ethics Committee for Research on Human Health (CRERSH/C) (No. 005777/CRERSHC). Written informed consent was obtained from all participants prior to their inclusion in the study. For illiterate participants, an oral explanation was provided in the local language in the presence of a witness, and a thumbprint was obtained as a substitute for written consent. Participation was voluntary, and data confidentiality was strictly maintained.

## 3. Results

### 3.1. Sociodemographic Characteristics

We recruited 422 newborns (aged 0–14 days) from postpartum and vaccination services at six health facilities. The mothers had an average age of 25.11 ± 4.98 years, with a mean parity of 2.01. Then, 46.7% lived alone, and 79.9% had completed secondary education. Most were Christian (87.91%), and 49.1% of the newborns were male. A large proportion of mothers (81.6%) earned less than 50,000 FCFA, and 62.5% had received breastfeeding initiation education before delivery. The sociodemographic data are shown in Table 1.

**Table 1:** Sociodemographic characteristics of mothers and newborns (*n* = 422) in the early initiation analysis, Bafia Health District, Cameroon.

Variable	Frequency (%)
Sex of newborn	
Male	207 (49.1)
Female	215 (50.9)
Marital status	
Alone	197 (46.7)
Couple	225 (53.3)
Level of education	
None	1 (0.2)
Primary	35 (8.3)
Secondary	337 (79.9)
University	49 (11.6)
Monthly income (FCFA)	
< 50,000	342 (81.6)
50,000–100,000	66 (15.8)
100,000–200,000	6 (1.4)
200,000–400,000	3 (0.7)
> 400,000	2 (0.5)
Multiple pregnancy	
Singleton	387 (91.7)
Multiple	35 (8.3)
Term pregnancy	
Premature	19 (4.5)
Term	402 (95.5)
Sensitization on breastfeeding initiation during pregnancy	
Yes	263 (62.5)
No	158 (37.5)
Mode of delivery	
Vaginal delivery	393 (93.1)
Cesarean section	26 (6.2)
Manual vacuum assistance	3 (0.7)

*Note:* Categorical variables are presented as frequencies and percentages. Continuous variables are presented as means ± standard deviation.

Abbreviations: CPN = antenatal consultation, FCFA = Central African CFA franc.

During our study across six health facilities in the Bafia Health District, we enrolled 422 newborns aged from birth to 14 days and 544 infants aged between 6 and 15 months. The mothers of the newborns had an average age of 25.11 years, while the mothers of the infants had a slightly higher average age of 26.22 years. Most mothers in both groups had completed secondary school (79.9% and 76.3%, respectively) and had a low monthly income of less than 50,000 FCFA (81.6% and 86.2%, respectively). The infants′ mothers mainly lived as part of a couple (60.7%), whereas 46.7% of the newborns′ mothers lived alone (Table 2).

**Table 2:** Sociodemographic characteristics comparison between mothers of newborns (*n* = 422) and mothers of infants aged 6–15 months (*n* = 544), Bafia Health District, Cameroon.

Variable	Infant group, *n* = 544, number (%)
Sex	
Male	291 (53.5)
Female	253 (46.5)
Marital status	
Single	214 (39.3)
Couple	330 (60.7)
Level of education	
None	3 (0.6)
Primary	78 (14.3)
Secondary	415 (76.3)
University	48 (8.8)
Monthly income (FCFA)	
< 50,000	469 (86.2)
50,000–100,000	60 (11.0)
100,000–200,000	12 (2.2)
200,000–400,000	2 (0.4)
> 400,000	1 (0.2)
Term pregnancy	
Premature	12 (2.2)
Term	532 (97.8)
Sensitization on EBF duration during pregnancy	
Yes	341 (62.7)
No	203 (37.3)
Mother’s HIV serology	
Positive	4 (0.7)
Negative	520 (95.6)
Unknown	20 (3.7)

*Note:* Data presented as frequencies (percentages) for categorical variables and means ± standard deviation for continuous variables.

### 3.2. Prevalence of EBF and Initiation Time

In the Bafia Health District, 73.7% (311/422) of mothers breastfed within 60 min of birth; 89.8% preferred breastfeeding immediately after birth; 53.9% exclusively breastfed for 6 months (see Table 3).

**Table 3:** Prevalence of breastfeeding practices in Bafia Health District, Cameroon.

Variable	Number (%)	95% CI
Timing of breastfeeding initiation after birth		
> 60 min	111 (26.3)	[22.33–30.70]
≤ 60 min	311 (73.7)	[69.30–77.67]
Breastfeeding method chosen at birth		
Exclusive breastfeeding	378 (89.8)	[86.52–92.33]
Mixed feeding	36 (8.6)	[6.24–11.61]
Artificial feeding	7 (1.7)	[0.81–3.39]
Length of exclusive breastfeeding (months)		
< 6 months	251 (46.1)	[41.99–50.34]
≥ 6 months	293 (53.9)	[49.66–58.01]

*Note:* Early initiation defined as breastfeeding within 60 min of birth. Exclusive breastfeeding (EBF) defined as feeding only breast milk without any other liquids or solids except vitamins, minerals, or medicines for the first 6 months of life. Data presented as frequencies (percentages) with 95% confidence intervals.

### 3.3. Factors Associated With Early Initiation of Breastfeeding

Maternal awareness during pregnancy about breastfeeding initiation (aOR = 2.22; *p* < 0.01), vaginal delivery (aOR = 5.64; *p* < 0.01), and singleton pregnancy (aOR = 2.31; *p* = 0.03) were independently associated with early breastfeeding initiation in multivariate analysis (Table 4).

**Table 4:** Factors associated with early initiation of breastfeeding (within 60 min of birth) among newborns in Bafia Health District, Cameroon (*n* = 422).

Variable	> 60 min	≤ 60 min	OR [95% CI]	*p* value	AOR [95% CI]
Sensitization on breastfeeding initiation during pregnancy					
Yes	55	208	2.08	< 0.01	2.22
			[1.36–3.22]		[1.34–3.67] ^∗^
No	56	102			
Mode of delivery					
Vaginal	92	301	6.17	5.64	
				[2.30–13.85] ^∗^	
			[2.66–14.32]	
Other (CS/MVA)	19	10			
Pregnancy type					
Singleton	96	291	2.27	0.02	2.31
			[1.12–4.62]		[1.07–4.95] ^∗^
Multiple	15	20			
Number of ANC visits					
≤ 4	70	230	0.60	0.02	1.56
			[0.38–0.95]		[0.96–2.53]
> 4	41	81			

*Note:* Results from multivariate binary logistic regression analysis. Variables included in the final model were selected based on univariate analysis (*p* < 0.25) and clinical relevance. Statistical significance set at *p* < 0.05.

Abbreviations: ANC = antenatal care, aOR = adjusted odds ratio, CI = confidence interval, CS = cesarean section, MVA = manual vacuum assistance.

^∗^Statistically significant (*p* < 0.05).

### 3.4. Factors Influencing EBF

Table 5 indicates that sensitization received during pregnancy (aOR = 2.67; *p* < 0.01), the absence of professional activity (aOR = 7.23; *p* < 0.01), the presence of maternity leave (aOR = 4.92; *p* < 0.01), the mother′s level of education (aOR = 2.04; *p* = 0.02), the antenatal care provider (aOR = 1.82; *p* = 0.03), and Muslim religion (aOR = 4.20; *p* < 0.01) were independently associated with EBF continuation.

**Table 5:** Factors associated with exclusive breastfeeding continuation for 6 months among infants in Bafia Health District, Cameroon (*n* = 544).

Variable	EBF < 6 months	EBF ≥ 6 months	OR (95% CI)	*p* value	AOR (95% CI)
Sensitization on EBF during pregnancy					
Yes	118	223	3.59 (2.49–5.17)	< 0.01	2.67 (1.72–4.15) ^∗^
No	133	70			
Absence of professional activity					
Yes	18	116	9.92 (5.80–16.98)	< 0.01	7.23 (6.83–16.28) ^∗^
No	231	150			
Presence of maternity leave					
Yes	7	40	6.11 (2.68–13.94)	< 0.01	4.92 (1.98–11.86) ^∗^
No	242	226			
Schooling					
Yes	198	265	2.53 (1.54–4.15)	<0.01	2.04 (1.07–3.89) ^∗^
No	53	28			
ANC provider					
Midwife	89	159	2.16 (1.53–3.05)	< 0.01	1.82 (1.29–2.96) ^∗^
Other	162	134			
Religion					
Muslim	9	25	2.58 (1.18–5.65)	0.01	4.20 (1.50–11.72) ^∗^
Christian	242	260			

*Note:* Results from multivariate binary logistic regression analysis. Variables with *p* < 0.25 in univariate analysis were included in the multivariate model. Statistical significance determined at *p* < 0.05.

Abbreviations: ANC = antenatal care, aOR = adjusted odds ratio, CI = confidence interval.

^∗^Statistically significant (*p* < 0.05).

## 4. Discussion

### 4.1. Prevalence of Exclusivity and Early Initiation Time

#### 4.1.1. Early Initiation of Breastfeeding

Our study shows that 73.7% of newborns were initiated to breast milk within the hour following birth. This result is higher than the national average of 48% reported in the 2018 DHS in Cameroon [9] and is also higher than rates reported in several African and non‐African countries, including Nigeria (59.2%), Senegal (3.75%), and India (41.5%) [8, 13, 14]. Our rate is consistent with rates reported in comparable sub‐Saharan African settings, such as 71.3% in Ethiopia [15] and 68.0% in Uganda [16], suggesting that health facility–based recruitment may capture a more health‐engaged population. This observed difference may be explained by the fact that our study population would have benefited from capacity building strategies and health professional support in the clinical monitoring system in reproductive health, which could have positively influenced early breastfeeding initiation practices.

#### 4.1.2. Exclusivity of Breastfeeding

We found that 53.9% of infants received exclusively breast milk during the first 6 months. This proportion is higher than the 40% found at the national level in the 2018 DHS [8]. This rate also exceeds the WHO target set in 2012, which aimed to bring the rate of EBF during the first 6 months to at least 50% by 2025 [17]. The practice of EBF in our study therefore surpasses both the national average and the global objective. Our result was higher than those obtained in comparable studies conducted in Senegal, Nigeria, and Ethiopia (51.3%, 52.9%, and 45.8%, respectively) [13, 18, 19].

The relatively high EBF prevalence observed may partly reflect a selection bias inherent to health facility–based recruitment, as mothers attending vaccination services and postnatal consultations may be more health‐engaged and receptive to breastfeeding promotion than the general population. Additionally, the fact that most mothers in our study were located in a semirural area and had no professional activities likely increased their availability for their infants. The relatively low economic level of most mothers also made it difficult to purchase breast milk substitutes. This should be taken into account when interpreting the generalizability of our results.

### 4.2. Study of Factors Associated With Early Initiation and Exclusivity of Breastfeeding

#### 4.2.1. Factors Associated With Early Initiation of Breastfeeding

Maternal awareness of breastfeeding practices during pregnancy was significantly associated with both early initiation and EBF, underscoring the importance of antenatal counselling in shaping postnatal breastfeeding behavior. The results of multivariate analysis found three independent factors associated with early initiation: sensitization received by mothers on the duration of initiation during pregnancy, vaginal delivery, and singleton pregnancy.

Our study shows that mothers who received sensitization on breastfeeding during pregnancy had twice the chance of having early initiation than those who had not received it. This was consistent with results from studies found in the literature [16, 20]. Prenatal visits or appointments serve as opportunities for mothers to be in contact with health professionals; several research studies have revealed that approaches such as health promotion and breastfeeding counselling during prenatal visits can improve the rate of early breastfeeding initiation [21]. Thus, intensification of prenatal intervention programs at the institutional level offers a good opportunity for mothers to acquire knowledge.

Mothers who delivered vaginally had nearly six times the odds of having early breastfeeding initiation compared to those who underwent cesarean section or assisted delivery. This result is similar to those obtained by other teams who also identified vaginal delivery as a determinant of early initiation [15, 21]. Cesarean section would delay early initiation of breastfeeding due to prolonged recovery after spinal anesthesia, postoperative maternal fatigue, postinterventional pain, uncomfortable breastfeeding position, decreased direct skin‐to‐skin contact, or insufficient breast milk production due to reduced oxytocin release following anesthesia [22].

We found in our study that singleton pregnancy was significantly associated with early breastfeeding initiation compared to multiple pregnancies. This result was consistent with those found in the literature [23]. This may be explained by the often‐delayed delivery of the placenta and the recurrence of maternal and neonatal complications observed in multiple pregnancies, which are generally associated with a higher frequency of prematurity and growth retardation, dystocia, and fetal distress, sometimes requiring cesarean section—situations that can initially delay early breastfeeding [24].

#### 4.2.2. Factors Associated With EBF

The results of multivariate analysis by logistic regression identified six independent factors associated with EBF: sensitization received by mothers on the duration of exclusivity during pregnancy, absence of maternal professional activities, presence of maternity leave, schooling, antenatal care provider, and Muslim religion.

Mothers who received sensitization during pregnancy were more likely to continue EBF than those who had not received it. This result suggests the importance of educating mothers about EBF. Several studies conducted in Senegal, Ethiopia, and Ecuador found a positive association between EBF practice and knowledge about breastfeeding [18, 19, 25]. A randomized trial conducted in Huzhu County in China found that mothers exposed to WeChat, a breastfeeding promotion application during pregnancy, continued EBF longer than those who had not used it [26]. Adam et al. in a cluster randomized controlled trial in South Africa drew similar conclusions [27]. These results highlight the responsibility of health professionals in educating mothers and the importance of policies promoting antenatal care visits.

The absence of maternal professional activity and the presence of maternity leave were factors significantly associated with the continuation of EBF. Mothers who work can be overwhelmed by their professional activities and generally have limited contact time with their infants. Setegn et al. in Ethiopia found that mothers without professional activities practiced EBF relatively better than their counterparts [28]. In Cameroon, the Labour Code (Law No. 92/007 of August 14, 1992) and the Social Insurance Code (Ordinance No. 73/17 of May 22, 1973) entitle employed mothers to a maternity leave of at least 14 weeks, with full pay for those affiliated to the National Social Insurance Fund (NSIF). However, a large proportion of women in Cameroon are engaged in informal employment and thus may not benefit from these statutory provisions, which may contribute to early cessation of EBF. The International Labour Organization in its Recommendation Number 191 regarding maternal protection provides for a maternity leave duration of at least 18 weeks and the possibility for the mother to choose the appropriate time for going on leave and the possibility of introducing daily breastfeeding breaks during professional activity [29].

Mothers who received school education had twice the chance of continuing EBF compared to those who had not received schooling. More educated mothers were more likely to practice EBF than mothers with little or no education [30]. Other studies conducted in Ethiopia drew similar conclusions [31, 32]. Mothers who had received schooling had greater ease in familiarizing themselves with new communication technologies and therefore informing themselves about the benefits and advantages of breast milk [32]. Along the same lines, Mekebo et al. found that women with higher education were more aware and receptive to the advantages of EBF [30].

Muslim religion was statistically associated with EBF practice compared to other religions. This difference may be explained by the fact that most Muslims consider breastfeeding as a right granted to the child according to Sharia law (Islamic law), and following the teachings of the Quran, Muslim mothers often aim to breastfeed their infants until the age of 2 years. Moreover, most Muslim mothers in our study were housewives, without professional activities and consequently more available to their infants.

## 5. Study Limitations

To our knowledge, this study is among the first conducted in the Bafia Health District in the field of breastfeeding, and as such could serve as a reference for future studies. We used logistic regression during analysis, which allows for mitigation of certain confounding factors. Nevertheless, the study has several limitations.

Memory bias may have been induced by some mothers forgetting the exact period of initiation or duration of EBF.

Selection bias: Our study included mother–infant pairs recruited from postnatal and vaccination services at second‐ and third‐category hospitals and did not include newborns and infants from satellite sites, private facilities, or the community, which limits the representativeness of our sample.

The results obtained in our study cannot be generalized to the entire country.

## 6. Conclusion

In summary, this study reveals that Bafia Health District exceeds Cameroon′s national averages with 73.7% early initiation and 53.9% EBF, yet remains below WHO targets. The key determinant factors—maternal pregnancy awareness (aOR = 2.67), vaginal delivery (aOR = 5.64), and lack of maternal employment (aOR = 7.23)—constitute priority intervention levers. Health systems must strengthen prenatal counselling programs and promote safe vaginal delivery, and policymakers must extend maternity leave while developing workplace lactation support policies. A coordinated approach targeting maternal education, improved obstetric practices, and workplace support could help achieve the WHO targets and significantly improve infant health outcomes in the region.

## Author Contributions

D.E., C.E.B., C.G.Y.E., and J.A. designed the study. D.E., C.E.B., and C.G.Y.E. collected the data. D.E., C.E.B., C.G.Y.E., and J.A. analyzed the data. D.E. wrote the first draft of the manuscript.

## Funding

No funding was received for this manuscript.

## Disclosure

All authors contributed to subsequent drafts and approved the final version.

## Conflicts of Interest

The authors declare no conflicts of interest.

## Supporting information


**Supporting Information** Additional supporting information can be found online in the Supporting Information section.

## Data Availability

The data that support the findings of this study are available from the corresponding author upon reasonable request.
